# Vascular Disease in Diabetic Women: Why Do They Miss the Female Protection?

**DOI:** 10.1155/2012/570598

**Published:** 2012-09-03

**Authors:** Ana Paula Villela Dantas, Zuleica Bruno Fortes, Maria Helena Catelli de Carvalho

**Affiliations:** ^1^Experimental Cardiology Division, Institut d'Investigacions Biomèdiques August Pi i Sunyer (IDIBAPS) and Institut Clínic de Tòrax, C/Casanova 143 Laboratory, IDIBAPS 404, Hospital Clinic, 08036 Barcelona, Spain; ^2^Laboratory of Hypertension, Department of Pharmacology, The Biomedical Sciences Institute, University of São Paulo, 2415 São Paulo, SP, Brazil

## Abstract

Gender plays a pivotal role in the onset as well as in the progression of the cardiovascular disease with a higher morbidity and mortality being detected in men with respect to women. Type 2 Diabetes Mellitus (T2DM) may reduce gender-related differences in the prevalence of cardiovascular disease by fading the vascular protective effects afforded by estrogen in females. This article will discuss the role of sex and sex hormones on the incidence and mechanisms involved in vascular dysfunction associated to T2DM, which might explain why women with T2DM lack the vascular protection.

## 1. The Basis of Diabetic Vascular Disease

Over the past decade type 2 diabetes mellitus (T2DM) has gained widespread attention among scientists and physicians because it has reached epidemic proportions in developed countries. The rapidly increasing prevalence and incidence of T2DM worldwide is likely a consequence of change in lifestyle patterns that contribute to obesity, and has become one of the most serious and challenging health problems in the 21st century. Beside endocrinologists, cardiologists are also meeting most of these patients since cardiovascular diseases (CVDs) are principal cause of morbidity and mortality in patients with T2DM. The detrimental manifestations to the vasculature include endothelial dysfunction and vascular inflammation, which, in turn, contributes to the high incidence of hypertension and atherosclerosis in those patients [1, 2].

**Figure 1:**
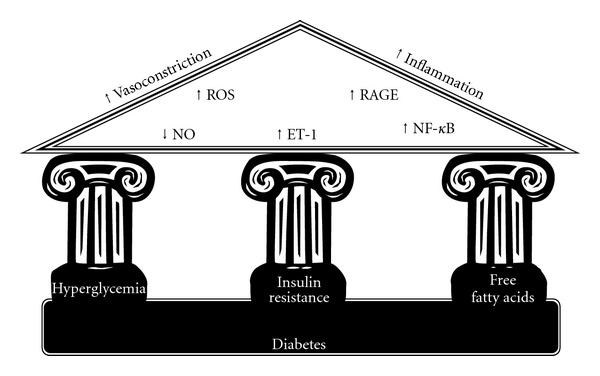
The three pillars of metabolic abnormalities that characterize diabetes and the molecular mechanism that can lead to diabetic vascular disease.

The major metabolic derangement during T2DM, that is, hyperglycemia, insulin resistance, and fatty acid liberation, has been considered the three pillars for diabetic vascular disease (Figure 1), as they evoke a myriad of molecular mechanisms that alter the structure and function of the vascular wall [1, 2]. These alterations include decrease of nitric oxide availability, increased oxidative stress, activation of the inflammation cascade, and of receptors for advanced glycation products (RAGE).

Initial studies on the pathophysiology of diabetic vascular disease have mostly associated vascular damage to hyperglycemia. In both clinical and experimental studies, hyperglycemia has been shown to enhance oxidative stress, to impair NO-mediated vasodilatation, and to initiate an inflammatory profile [1, 2]. The role of high glucose levels to vascular damage is supported by the observation that glycemia restoration with insulin is capable to restore vascular reactivity of diabetic subjects [3–5]. Hyperglycemia may initiate vascular dysfunction by directly activating mitochondrial electron transport and increasing superoxide production. Moreover, hyperglycemia has been described to directly impair NO/superoxide systems via an increase of asymmetric dymethylarginine (ADMA), a competitive antagonist of NO synthase [1, 2]. Further long-term hyperglycemia contributes to vascular disease through the intracellular and extracellular formation of advanced glycation end products (AGEs), a group of molecules (proteins, lipids, and nucleic acids) that are irreversibly cross-linked with reducing sugars. AGEs are involved in the process of vascular dysfunction directly or via receptor-mediated mechanisms [6]. The interaction of AGEs with its receptors (RAGE) triggers a variety of cellular signaling that mediate gene expression and enhances the release of proinflammatory molecules and oxidative stress [7]. RAGE activation results in the translocation of proinflammatory kinases and transcription factors including extracellular signal-related (ERK) and c-Jun N-terminal (JNK) mitogenactivated protein (MAP) kinases, and the proinflammatory transcription factor nuclear Factor-*κ*B (NF-*κ*B) [7, 8]. Activation of these molecules has been tightly linked to the upregulation of inflammatory markers, including tumor necrosis factor (TNF*α*), and interleukins (IL-6), and adhesion molecules (such as VCAM-1 and ICAM-1), and to activation of prooxidative pathways [9]. 

Despite the undeniable role of hyperglycemia to the diabetic vascular disease, it is important highlight that individuals with T2DM may display signs of endothelial dysfunction and vascular inflammation even before the development of clinical manifestations of hyperglycemia [10]. This theory is supported by studies that demonstrated abnormal vascular reactivity of nondiabetic siblings and children of patients with T2DM [11]. Although they were nondiabetic, those who exhibited alterations of the vascular response had some degree of insulin resistance. In physiological conditions, insulin promotes endothelium-dependent relaxation, by a mechanism that involves increase of NO production via activation of phosphatidylinositol-3 kinase (PI3 K) and Akt kinase pathways [12, 13]. In insulin-resistant individuals, however, endothelium-dependent relaxation and NO production by insulin are reduced or even null [14–16]. When insulin signal transduction is impaired and insulin is less able to activate NO via PI3 K/Akt pathways, there is a deviation from this pathway to activate the mitogen-activated protein kinase (MAPK) pathway [17]. The MAPK pathway is well known for its proliferative actions in the smooth muscle cells. Also, MAPK activation is associated with increased endothelin-1 production and at greater extent to activation of inflammation [18, 19]. These observations have many implications that correlate insulin signaling with vascular dysfunction in T2DM, as a consequence of malfunctioning of insulin signaling pathways. In this regard, in a state of insulin resistance, insulin itself may contribute to accelerated vascular damage as it may display proatherogenic and prohypertensive potentials.

Circulating levels of free fatty acids have also been gaining special protagonism in the pathophysiology of diabetic vascular disease, not only because of their excessive liberation from adipose tissue but also because obesity has been tightly linked to insulin resistance and T2DM [20]. Increased body fat, as seen in T2DM and insulin resistance, causes increased lipolysis and increased circulating concentrations of nonesterified fatty acids, as well as other components that are key mediators of vascular dysfunction, including angiotensinogen, adiponectin, IL-6, prostaglandins, and TNF*α* [21, 22]. Emerging evidences have established that adiponectin, an adipocyte-derived protein, plays a key role in many metabolic derangements, including type 2 diabetes, through its involvement in glucose regulation and fatty acid catabolism. Longitudinal and cross-sectional studies have shown that adiponectin concentration negatively correlates with the development of insulin resistance and predict the progression of type 2 diabetes and are associated with a variety of human metabolic and cardiovascular disease states, including obesity, essential hypertension, and coronary artery diseases [23]. In addition, in vitro and in vivo studies have shown that exogenous administration of free fatty acids can alter the function of endothelial cells so as to create a profile which promotes vasoconstriction and vascular inflammation [22, 24, 25]. Recently, a translational study has elegantly demonstrated that endothelial cells grown in the presence of visceral secretomes from obese and insulin-resistant patients display increased proliferation, altered morphology, and augmented expression of adhesion molecules (VCAM-1 and ICAM-1), and higher reactivity towards circulating platelets [26]. These changes occurred through a mechanism that involves NF-*κ*B activation, largely described in the literature as major mediator of vascular damage in T2DM [9].

## 2. Gender and Risk Factors for Cardiovascular Disease

Experimental and clinical studies support the hypothesis that men are hemodynamically older than age-matched, premenopausal women [27–29]. According to two major longitudinal studies—the Framingham Heart Study [30] and the INTERHEART [31, 32]—the overall median age for evident CVD is about 10 years lower in men than in their female counterparts, in all regions of the world. Also in animal models, the progression of CVD occurs at an earlier age and becomes more severe in males compared to age-matched females [33–35]. The female protection might be a consequence of women that have been exposed to lower and less severe risk factors for CVD. In fact, gender-associated differences have been noted in the pathophysiology of most major risk factors, including hypertension and atherosclerosis. 

High blood pressure is a global health concern reaching the number of more than one billion diagnosed patients worldwide [36]. Isolated hypertension, defined as a systolic blood pressure ≥160 mmHg and a diastolic blood pressure <90 mmHg, is associated with an increased risk of cardiovascular disease, stroke, and all-cause mortality both in men and women independent of other risk factors [37]. Ambulatory monitoring of blood pressure have shown a sexual dimorphism in the incidence of high blood pressure that becomes apparent prematurely during adolescence and persists throughout adulthood [38]. Moreover, several cross-sectional studies have described that, up to middle age, men had a higher prevalence of hypertension than women regardless of race and ethnicity [39–41]. After the age of 65, however, women have higher prevalence of hypertension across all racial and ethnic groups [39–41]. The INTERHEART study has described a greater risk for CVD associated with hypertension in women than in men partially explained by a higher prevalence of hypertension in women who were about a decade older than hypertensive men [32]. The protective effects of female gender seen in humans have also been observed in various animal models for cardiovascular disease, such as spontaneously hypertensive rats (SHRs) and DOCA-salt hypertensive rats [42]. In these animals, males develop an earlier and more severe cardiovascular disease than females.

Besides hypertension, gender-associated differences in the incidence and progress of atherosclerotic plaque have also been proposed. Atherosclerosis is the leading cause of heart attack, and despite its high incidence and severity, there is still a concerning lack of studies addressing the incidence and risks of atherosclerosis in women. A recent observation at autopsy of patients who died from acute coronary disease has described a “gender gap” on vascular inflammation and atherosclerosis formation [43]. According to this study, inflammatory atherosclerosis and associated acute coronary heart disease develop earlier in life in men than in women, and they are associated with death at an earlier age, although both men and women present the same overall plaque burden [43]. Recently, the PROSPECT study has described that women have less extensive coronary artery disease, and that atherosclerotic lesions in women compared with men, have less plaque rupture and less necrotic core and calcium, despite similar plaque burden [44]. Also, in animal models for atherosclerosis, male gender contributes to the progression of lipid deposition, remodeling, and aortic lesions [45–47]. 

In addition, cigarette smoking is one of the most powerful risk factor predisposing to cardiovascular disease. Smoking causes more deaths from coronary heart disease and stroke than any other cigarette-associated disease in both men and women [48]. Despite its evident risk, to date, few studies have addressed to the sex-relative importance of smoking as a risk factor for fatal and nonfatal cardiovascular diseases within the same study population. On the basis of available clinical data, smoking has been identified as a stronger risk factor in women in comparison to men (relative risk 3.3 versus 1.9), which become more pronounced when considering younger women under 45 years of age [48]. In this particular population, cigarette smoking is clearly the most important risk factor for sudden cardiac death [49]. Cigarette smoking has been strongly associated with atherosclerotic complications as well as increased risk for acute coronary thrombosis. In fact, acute myocardial infarction among smokers is most often precipitated by thrombosis than unstable atherosclerotic lesions [48]. In this regard, the fact that female gender and associated factors—contraceptive use, hormone replacement therapy, and pregnancy—are linked to increased risk of thrombosis could plausibly explain why female smokers are at greater risk. 

Despite all evidences, however, there are still some discrepancies between studies in demonstrating a sex difference of smoking as a risk factor for cardiovascular disease. For instance, the Framingham study failed to demonstrate a positive correlation between smoking and coronary heart disease among women [50, 51]; and in some American [52] and British [53] studies for coronary mortality, the relative risks associated with smoking were similar when comparing both genders. These conflicting results may be related to several factors such as sex differences in smoking habits and cessation during followup, different age distribution of men and women included into the studies, or use of oral contraceptive use and hormone replacement therapy. Due to the amount of variables that may influence the detrimental effects carried by tobacco use, the sex-relative cardiovascular risk associated with cigarette smoke should be interpreted cautiously. 

When it comes to diabetes, however, a general consensus has validated that women are at greater risk for CVD than man, even though the incidence of DM is found to be similar in women and men. In fact, the Nurses' Health Study found CVD mortality in women with DM to be 8.7 times higher than in nondiabetic female patients [54]. The INTERHEART study of risk factors for CVD identified diabetes mellitus as one of the greatest risk factor for women, as diabetic women had a threefold to fourfold increased risk of developing CVD compared to men [32], and a recent meta-analysis of 37 studies consisting of almost 450,000 patients with type 2 diabetes found that women have a threefold increased risk of fatal coronary heart disease, whereas men have a twofold increased risk [55]. The variance in the phenomenon remains to be elucidated and contrary to other risk factors for CVD, the gender-associated differences in experimental model of diabetes do not reflect what is seen in humans [56]. 

Most pathophysiological studies on T2DM have been performed in rodents and in the majority of experimental models males are more susceptible to develop T2DM and are more vulnerable to its vascular complications than are females [56]. In general, diabetic male are found to have worse endothelial-dependent relaxation, augmented vasoconstrictor responses, and higher blood pressure levels than do females [15, 57, 58]. Just few studies have shown that T2DM impairs endothelial responses in female to a greater extent than in males [59]. These discrepant data may be a consequence of distinct etiopathology for T2DM in each model. In spontaneous or diet-induced diabetes, some models exhibit a predominant insulin resistance, while in others glucose, intolerance is a part of a wider phenotype of adiposity [60]. Other models have been generated from genetic manipulations for the ablation of the genes involved in insulin pathway [61]. Although the existing models offer many opportunities to investigate the complex mechanisms of T2DM-associated vascular disease, no individual animal model replicates in all details the progression of human T2DM. Besides, variations in the hormonal regulation that are characteristic of each species can lead to confounding and misleading outcomes, since several sex-associated differences in the control of vascular function are partially sustained by sexual hormones [33, 62–65]. 

## 3. Sex Hormones and the Pillars for Diabetic Vascular Disease 

The differences in T2DM burden in men and women could be explained by the differences in socioeconomic status between the two genders. As women tend to have lower economical status than men they could be at greater risk of developing T2DM as well as to T2DM-associated complications to have lower access to treatments for glucose control and to prevent vascular dysfunction. In fact, as per the World Health Organization (WHO), the estimate prevalence of diabetes and other abnormalities of glucose metabolism is consistent across income grouping worldwide, although these differences do not vary among sexes [66]. From a physiological standpoint, epidemiological observations and extensive basic laboratory research have shown that female sex hormones, and more specifically estrogen, have direct beneficial effects in the cardiovascular system [64, 65, 67–69]. Estrogen has been described to display a myriad of metabolic, hemodynamic, and vascular effects, which have been largely associated to cardiovascular protection in females. For example, estrogen can promote cardiovascular protection by indirectly influence on the metabolism of lipoproteins or directly by acting on the modulation of molecular pathways in the vessel wall [70]. Receptors for estrogen have been identified biochemically and show a plentiful expression in both vascular smooth muscle and endothelium, reinforcing the idea that estrogen plays a key role in the control of vascular function [71, 72].

Other studies have described that estrogen has direct beneficial effects in the control of blood pressure [65, 67, 69, 73] and decrease of atheroma formation [74–77]. Although the mechanisms underlying the protective effects of estrogen in the vasculature are not well established, a direct regulation of endothelial-mediated control of arteriolar tone and during different stages of development of atherosclerosis has been proposed [42, 70]. Estrogen is known to increase NO bioavailability by mechanisms that involve either increase of NO generation directly [78] or by decreasing O_2_^−^ concentration, and thereby attenuating O_2_^−^-mediated inactivation of NO [42, 68]. In addition to NO, estrogen has been described to positively up regulate the production of endothelium-derived relaxing factors (EDRFs), such as PGI_2_ [79, 80] and the endothelium-derived hyperpolarizing factors (EDHFs) [81], both of which are important mediators of vascular relaxation in resistance-sized arteries. Concomitantly, a modulating role of estrogen on constrictor factors (EDCFs) is observed. Studies have shown that the beneficial effects of estrogen on the endothelium can be partially explained by an inhibitory effect on the production or action of the COX-derived vasoconstrictor agents (PGH_2_ and TXA_2_) [65, 82, 83] and endothelin-1 (ET-1) [84]. Estrogen has also been described to suppresses vascular inflammation by downregulation of proinflammatory molecules including cytokines and adhesion molecules [85–90].

When considering the modulation of the metabolic changes that build the pillars for diabetic vascular disease, estrogen is a major effector for the regulation of energy balance, fat distribution, and insulin sensitivity [91]. Postmenopausal women and ovariectomized females experience an increase in fat mass and insulin resistance, which can be reversed by estrogen [91]. In this regard, a protective response by estrogen should be expected in T2DM women. In fact, a variety of studies in animals models have confirmed the protective effects of estrogen against diabetes [92], and one of the most renowned trial on women's health and hormone replacement therapy—the Women's Health Initiative (WHI)—has shown positive correlation between daily estrogen treatment over placebo on different parameters of diabetes, including blood glucose, insulin, calculated insulin resistance, and the self-reported incidence of diabetes. Results over more than 5 years of followup revealed that therapy with estrogen reduces the incidence of diabetes, possibly mediated by mechanism that involves decrease in insulin resistance [93, 94]. Nonetheless, data from the same WHI study have questioned the value of estrogen replacement therapy in protecting vascular function [95]. The WHI trial did not find any cardiovascular benefit from estrogen in postmenopausal women and, in fact, showed hormone replacement therapy could be associated with increased risk to the cardiovascular system [95]. Further analysis by subgroups in those clinical trials has established that estrogen replacement therapy in diabetic postmenopausal women results in a seemingly detrimental effect on the cardiovascular system [96, 97]. With these striking results, the question arises as how and why estrogen responses are modified by diabetes state in women.

## 4. Why Are Diabetic Females Not Protected? 

Initial hypotheses relied on the hormonal dysfunction resulting from diabetic state to explain why women with diabetes lose their vascular protection [59, 98]. Others have associated increased risk for diabetes and associated vascular disease to estrogen deficiency after menopause, as the decline in estrogens levels often leads to dysregulation of metabolism [99]. Nonetheless, the use of estrogen replacement therapy has failed to decrease CVD risk in diabetic women, despite their improved metabolic outcomes [96, 97]. 

There is much controversy over the interpretation of the clinical trials on estrogen replacement therapy, and among the concerns raised is the fact that the majority of clinical trial on hormone replacement therapy, which studied a population of women that was estrogen deficient for, on average, 10 years before hormone replacement was initiated, and they may exhibit some degree of subclinical vascular dysfunction. These observations, together with observational studies, have led scientists to create the so-called timing hypothesis. This theory states that estrogen-mediated benefits to prevent cardiovascular disease only occur when treatment is initiated before the detrimental effects of long-term estrogen withdrawn or subclinical vascular damage are established on vascular wall [100]. 

**Figure 2:**
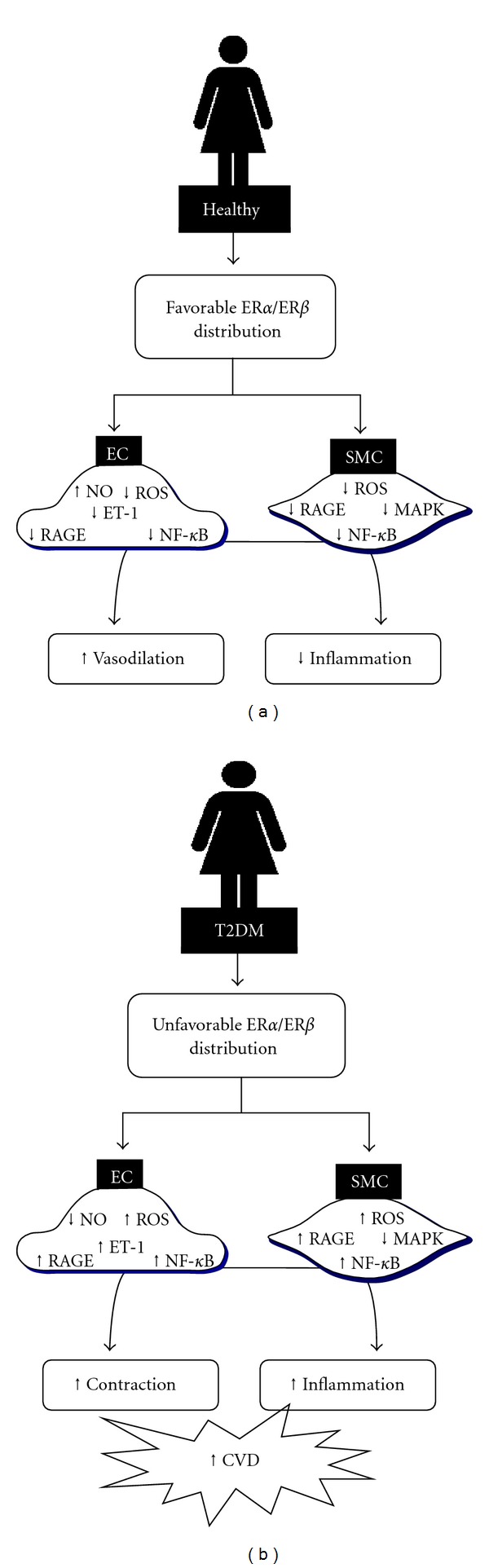
Hypothesis for detrimental estrogen responses in the diabetic vasculature: Type 2 diabetes mellitus-(T2DM-) related changes in the vessel wall include decrease of nitric oxide (NO) and concomitant increase of reactive oxygen species (ROS) and endothelin-1 (ET-1) production; as well as increased activation of signaling pathways of Nuclear Factor-*κ*B (NF-*κ*B); mitogen-activated protein kinases (MAPK) and receptors for advanced glycation products (RAGE). In a healthy vasculature (a), with favorable balance of estrogen receptors (ER), estrogen beneficially acts to modulate these factors and to maintain homeostasis. Nevertheless, T2DM adversely modify the balance in expression and/or activity of ERs in a manner that the effects of estrogen are negatively modulated to enhance the existing damage in vascular function (b).

Currently, it is not known how the vascular effects of estrogen may be influenced by distinct pathophysiological conditions, including aging or diabetes, but recent consensus have established a relationship between changes on balance of estrogen receptors (ER*α* and ER*β*) with dichotomous effects by estrogen (Figure 2). The differences in signaling through ER*α* and ER*β* are increasingly becoming apparent, and, in fact, previous experimental studies have established that increased expression or activation of ER*β* over ER*α* is associated with higher oxidative stress, proinflammatory profile and increased atherosclerotic plaque formation [101–104]. In animal model of diabetes, the anti-inflammatory activity of estrogen is impaired in vascular smooth muscle cells which display ER*β* overexpression with respect to normoglycemic controls [105, 106]. 

Results from studies using knockout mice for ERs have shed much light into their specific role to metabolic homeostasis and vascular function. While intact ER*α* knockout mice are diabetogenic and obese with severe insulin resistance, ovariectomized mice display a normal homeostasis of circulating glucose and insulin levels and reverses the obese phenotype, suggesting that estrogen may act on ER*β* to result in a diabetogenic and adipogenic phenotype [107]. Furthermore, the use of ER*β*-selective agonists has shown to be diabetogenic and to display a proinflammatory profile in diabetic animals [106, 108]. Despite those evidences, the field lacks detailed research as to how ER*α* and ER*β* affect the course and timeline of diabetic vascular disease. It remains unclear to what extent the protective effects of estrogen replacement well described in young health females can be extrapolated to older and diabetic ones. The mechanism for diabetic vascular disease in women issue still needs to be addressed in both experimental and clinical studies in order to establish different strategies to prevent delay or attenuate the vascular detriment induced by diabetes. 

## 5. Conclusions

This review calls attention to the lack in knowledge, understanding, and general awareness of medical and scientific societies on how to treat and prevent diabetic vascular disease in women. Despite the evident gender-associated differences in the phathophysiology of CVD and the higher incidence of vascular disease in diabetic women, the trends and guidelines are dominated by data obtained in men. The lack of crucial information from clinical trials and the discrepancies between the data available on the regulation of the cardiovascular system of women often leads to inappropriate diagnosis and specific treatment of this patient group, and, therefore, women are still not benefiting equally from effective risk-prevention strategies against CVD. Much research is still needed to ascertain and incorporate the gender-specific risks into the clinic to optimize diagnosis, treatment, and earlier prevention of CVD in women. 

## References

[1] Creager MA, Lüscher TF, Cosentino F, Beckman JA (2003). Diabetes and vascular disease. Pathophysiology, clinical consequences, and medical therapy: part I. *Circulation*.

[2] Lüscher TF, Creager MA, Beckman JA, Cosentino F (2003). Diabetes and vascular disease. Pathophysiology, clinical consequences, and medical therapy: part II. *Circulation*.

[3] Akamine EH, Kawamoto EM, Scavone C (2006). Correction of endothelial dysfunction in diabetic female rats by tetrahydrobiopterin and chronic insulin. *Journal of Vascular Research*.

[4] Rastelli VMF, Akamine EH, Oliveira MA (2005). Influence of insulin on the microvascular response to inflammatory mediators in neonatal streptozotocin diabetic rats. *Inflammation Research*.

[5] Schindler TH, Facta AD, Prior JO (2007). Improvement in coronary vascular dysfunction produced with euglycaemic control in patients with type 2 diabetes. *Heart*.

[6] Yamagishi SI, Maeda S, Matsui T, Ueda S, Fukami K, Okuda S (2011). Role of advanced glycation end products (AGEs) and oxidative stress in vascular complications in diabetes. *Biochimica et Biophysica Acta*.

[7] Farmer DGS, Kennedy S (2009). RAGE, vascular tone and vascular disease. *Pharmacology and Therapeutics*.

[8] Lander HM, Tauras JM, Ogiste JS, Hori O, Moss RA, Schmidt AM (1997). Activation of the receptor for advanced glycation end products triggers a p21(ras)-dependent mitogen-activated protein kinase pathway regulated by oxidant stress. *The Journal of Biological Chemistry*.

[9] Rial NS, Choi K, Nguyen T, Snyder B, Slepian MJ (2012). Nuclear factor kappa B, (NF-*κ*B): a novel cause for diabetes, coronary artery disease and cancer initiation and promotion?. *Medical Hypotheses*.

[10] Hsueh WA, Lyon CJ, Quiñones MJ (2004). Insulin resistance and the endothelium. *American Journal of Medicine*.

[11] Caballero AE (2003). Endothelial dysfunction in obesity and insulin resistance: a road to diabetes and heart disease. *Obesity Research*.

[12] Zeng G, Nystrom FH, Ravichandran LV (2000). Roles for insulin receptor, PI3-kinase, and Akt in insulin-signaling pathways related to production of nitric oxide in human vascular endothelial cells. *Circulation*.

[13] Zeng G, Quon MJ (1996). Insulin-stimulated production of nitric oxide is inhibited by Wortmannin: direct measurement in vascular endothelial cells. *Journal of Clinical Investigation*.

[14] Wang CCL, Goalstone ML, Draznin B (2004). Molecular mechanisms of insulin resistance that impact cardiovascular biology. *Diabetes*.

[15] Taguchi K, Matsumoto T, Kamata K, Kobayashi T (2012). Akt/eNOS pathway activation in endothelium-dependent relaxation is preserved in aortas from female, but not from male, type 2 diabetic mice. *Pharmacological Research*.

[16] Taguchi K, Kobayashi T, Matsumoto T, Kamata K (2011). Dysfunction of endothelium-dependent relaxation to insulin via PKC-mediated GRK2/Akt activation in aortas of ob/ob mice. *American Journal of Physiology—Heart and Circulatory Physiology*.

[17] Montagnani M, Golovchenko I, Kim I (2002). Inhibition of phosphatidylinositol 3-kinase enhances mitogenic actions of insulin in endothelial cells. *The Journal of Biological Chemistry*.

[18] Gogg S, Smith U, Jansson PA (2009). Increased MAPK activation and impaired insulin signaling in subcutaneous microvascular endothelial cells in type 2 diabetes: the role of endothelin-1. *Diabetes*.

[19] Oliver FJ, de la Rubia G, Feener EP (1991). Stimulation of endothelin-1 gene expression by insulin in endothelial cells. *The Journal of Biological Chemistry*.

[20] Roos CJ, Quax PH, Jukema JW (2012). Cardiovascular metabolic syndrome: mediators involved in the pathophysiology from obesity to coronary heart disease. *Biomarkers in Medicine*.

[21] Gomes F, Telo DF, Souza HP, Nicolau JC, Halpern A, Serrano CV (2010). Obesity and coronary artery disease: role of vascular inflammation. *Arquivos Brasileiros de Cardiologia*.

[22] de Carvalho MH, Colaco AL, Fortes ZB (2006). Cytokines, endothelial dysfunction, and insulin resistance. *Arquivos Brasileiros de Endocrinologia & Metabologia*.

[23] Swarbrick MM, Havel PJ (2008). Physiological, pharmacological, and nutritional regulation of circulating adiponectin concentrations in humans. *Metabolic Syndrome and Related Disorders*.

[24] Lobato NS, Filgueira FP, Akamine EH, Tostes RC, Carvalho MH (2012). Mechanisms of endothelial dysfunction in obesity-associated hypertension. *Brazilian Journal of Medical and Biological Research*.

[25] Maenhaut N, van de Voorde J (2011). Regulation of vascular tone by adipocytes. *BMC Medicine*.

[26] Hanzu FA, Palomo M, Kalko SG (2011). Translational evidence of endothelial damage in obese individuals: inflammatory and prothrombotic responses. *Journal of Thrombosis and Haemostasis*.

[27] Messerli FH, Garavaglia GE, Schmieder RE (1987). Disparate cardiovascular findings in men and women with essential hypertension. *Annals of Internal Medicine*.

[28] Bairey Merz CN, Shaw LJ, Reis SE (2006). Insights from the NHLBI-sponsored women’s ischemia syndrome evaluation (WISE) study. Part II: gender differences in presentation, diagnosis, and outcome with regard to gender-based pathophysiology of atherosclerosis and macrovascular and microvascular coronary disease. *Journal of the American College of Cardiology*.

[29] Shaw LJ, Bairey Merz CN, Pepine CJ (2006). Insights from the NHLBI-sponsored women’s ischemia syndrome evaluation (WISE) study. Part I: gender differences in traditional and novel risk factors, symptom evaluation, and gender-optimized diagnostic strategies. *Journal of the American College of Cardiology*.

[30] Pencina MJ, D’Agostino RB, Larson MG, Massaro JM, Vasan RS (2009). Predicting the 30-year risk of cardiovascular disease: the Framingham heart study. *Circulation*.

[31] Anand SS, Islam S, Rosengren A (2008). Risk factors for myocardial infarction in women and men: insights from the INTERHEART study. *European Heart Journal*.

[32] Yusuf S, Hawken S, Ounpuu S (2004). Effect of potentially modifiable risk factors associated with myocardial infarction in 52 countries (the INTERHEART study): case-control study. *The Lancet*.

[33] Chen YF, Meng QC (1991). Sexual dimorphism of blood pressure in spontaneously hypertensive rats is androgen dependent. *Life Sciences*.

[34] Ouchi Y, Share L, Crofton JT (1987). Sex difference in the development of deoxycorticosterone-salt hypertension in the rat. *Hypertension*.

[35] Rowland NE, Fregly MJ (1992). Role of gonadal hormones in hypertension in the Dahl salt-sensitive rat. *Clinical and Experimental Hypertension—Part A*.

[36] Kearney PM, Whelton M, Reynolds K, Muntner P, Whelton PK, He J (2005). Global burden of hypertension: analysis of worldwide data. *The Lancet*.

[37] D’Agostino RB, Vasan RS, Pencina MJ (2008). General cardiovascular risk profile for use in primary care: the Framingham heart study. *Circulation*.

[38] Burt VL, Whelton P, Roccella EJ (1995). Prevalence of hypertension in the US adult population: results from the third national health and nutrition examination survey, 1988–1991. *Hypertension*.

[39] Stamler J, Stamler R, Riedlinger WF, Algera G, Roberts RH (1976). Hypertension screening of 1 million Americans. Community hypertension evaluation clinic (CHEC) program, 1973 through 1975. *The Journal of the American Medical Association*.

[40] Flegal KM, Ogden CL, Carroll MD (2004). Prevalence and trends in overweight in Mexican-American adults and children. *Nutrition Reviews*.

[41] Cutler JA, Sorlie PD, Wolz M, Thom T, Fields LE, Roccella EJ (2008). Trends in hypertension prevalence, awareness, treatment, and control rates in United States adults between 1988–1994 and 1999–2004. *Hypertension*.

[42] Tostes RC, Nigro D, Fortes ZB, Carvalho MHC (2003). Effects of estrogen on the vascular system. *Brazilian Journal of Medical and Biological Research*.

[43] Frink RJ (2009). Gender gap, inflammation and acute coronary disease: are women resistant to atheroma growth? Observations at autopsy. *Journal of Invasive Cardiology*.

[44] Lansky AJ, Ng VG, Maehara A, Weisz G, Lerman A (2012). Gender and the extent of coronary atherosclerosis, plaque composition, and clinical outcomes in acute coronary syndromes. *JACC: Cardiovasc Imaging*.

[45] Pereira TMC, Nogueira BV, Lima LCF (2010). Cardiac and vascular changes in elderly atherosclerotic mice: the influence of gender. *Lipids in Health and Disease*.

[46] Surra JC, Guillén N, Arbonés-Mainar JM (2010). Sex as a profound modifier of atherosclerotic lesion development in apolipoprotein E-deficient mice with different genetic backgrounds. *Journal of Atherosclerosis and Thrombosis*.

[47] Holm P, Andersen HL, Arrøe G, Stender S (1998). Gender gap in aortic cholesterol accumulation in cholesterol-clamped rabbits: role of the endothelium and mononuclear-endothelial cell interaction. *Circulation*.

[48] Bolego C, Poli A, Paoletti R (2002). Smoking and gender. *Cardiovascular Research*.

[49] Oparil S (1998). Pathophysiology of sudden coronary death in women: implications for prevention. *Circulation*.

[50] Seltzer CC (1991). The negative association in women between cigarette smoking and uncomplicated angina pectoris in the Framingham heart study data. *Journal of Clinical Epidemiology*.

[51] Seltzer CC (1989). Framingham study data and “established wisdom” about cigarette smoking and coronary heart disease. *Journal of Clinical Epidemiology*.

[52] Hammond EC (1966). Smoking in relation to the death rates of one million men and women. *National Cancer Institute Monograph*.

[53] Doll R, Peto R, Wheatley K, Gray R, Sutherland I (1994). Mortality in relation to smoking: 40 years’ observations on male British doctors. *British Medical Journal*.

[54] Tanasescu M, Cho E, Manson JE, Hu FB (2004). Dietary fat and cholesterol and the risk of cardiovascular disease among women with type 2 diabetes. *American Journal of Clinical Nutrition*.

[55] Huxley R, Barzi F, Woodward M (2006). Excess risk of fatal coronary heart disease associated with diabetes in men and women: meta-analysis of 37 prospective cohort studies. *British Medical Journal*.

[56] Franconi F, Seghieri G, Canu S, Straface E, Campesi I, Malorni W (2008). Are the available experimental models of type 2 diabetes appropriate for a gender perspective?. *Pharmacological Research*.

[57] Takenouchi Y, Kobayashi T, Taguchi K, Matsumoto T, Kamata K (2009). Gender differences in endothelial function in aortas from type 2 diabetic model mice. *Journal of Pharmacological Sciences*.

[58] Takenouchi Y, Kobayashi T, Taguchi K, Matsumoto T, Kamata K (2010). Gender differences in vascular reactivity of aortas from streptozotocin-induced diabetic mice. *Biological and Pharmaceutical Bulletin*.

[59] Toledo DP, Akamine E, Nigro D, Passaglia RCT, Carvalho MHC, Fortes ZB (2003). Microvascular reactivity in experimental diabetes: responses of male and female rats. *Inflammation Research*.

[60] Rees DA, Alcolado JC (2005). Animal models of diabetes mellitus. *Diabetic Medicine*.

[61] Plum L, Wunderlich FT, Baudler S, Krone W, Brüning JC (2005). Transgenic and knockout mice in diabetes research: novel insights into pathophysiology, limitations, and perspectives. *Physiology*.

[62] Teede HJ (2007). Sex hormones and the cardiovascular system: effects on arterial function in women. *Clinical and Experimental Pharmacology and Physiology*.

[63] Silva-Antonialli MM, Fortes ZB, Carvalho MHC, Scivoletto R, Nigro D (2000). Sexual dimorphism in the response of thoracic aorta from SHRs to losartan. *General Pharmacology: The Vascular System*.

[64] Dantas APV, Franco MDCP, Tostes RCA (2004). Relative contribution of estrogen withdrawal and gonadotropins increase secondary to ovariectomy on prostaglandin generation in mesenteric microvessels. *Journal of Cardiovascular Pharmacology*.

[65] Dantas APV, Scivoletto R, Fortes ZB, Nigro D, Carvalho MHC (1999). Influence of female sex hormones on endothelium-derived vasoconstrictor prostanoid generation in microvessels of spontaneously hypertensive rats. *Hypertension*.

[66] WHO (2011). Burden: mortality, morbidity and risk factors. *Global Status Report on Noncommunicable Diseases 2010*.

[67] Staessen J, Bulpitt CJ, Fagard R, Lijnen P, Amery A (1989). The influence of menopause on blood pressure. *Journal of Human Hypertension*.

[68] Dantas APV, Tostes RCA, Fortes ZB, Costa SG, Nigro D, Carvalho MHC (2002). In vivo evidence for antioxidant potential of estrogen in microvessels of female spontaneously hypertensive rats. *Hypertension*.

[69] Hinojosa-Laborde C, Craig T, Zheng W, Ji H, Haywood JR, Sandberg K (2004). Ovariectomy augments hypertension in aging female Dahl salt-sensitive rats. *Hypertension*.

[70] Miller VM, Duckles SP (2008). Vascular actions of estrogens: functional implications. *Pharmacological Reviews*.

[71] Couse JF, Lindzey J, Grandien K, Gustafsson JA, Korach KS (1997). Tissue distribution and quantitative analysis of estrogen receptor-*α* (ER*α*) and estrogen receptor-*β* (ER*β*) messenger ribonucleic acid in the wild-type and ER*α*-knockout mouse. *Endocrinology*.

[72] Pau CY, Pau KYF, Spies HG (1998). Putative estrogen receptor *β* and *α* mRNA expression in male and female rhesus macaques. *Molecular and Cellular Endocrinology*.

[73] The Writing Group for the PT, Miller VT, LaRosa J, Barnabei V, Kessler C (1995). Effects of estrogen or estrogen/progestin regimens on heart disease risk factors in postmenopausal women: the postmenopausal estrogen/progestin interventions (PEPI) trial. *The Journal of the American Medical Association*.

[74] Tolbert T, Thompson JA, Bouchard P, Oparil S (2001). Estrogen-induced vasoprotection is independent of inducible nitric oxide synthase expression: evidence from the mouse carotid artery ligation model. *Circulation*.

[75] Chandrasekar B, Sirois MG, Geoffroy P, Lauzier D, Nattel S, Tanguay JF (2005). Local delivery of 17*β*-estradiol improves reendothelialization and decreases inflammation after coronary stenting in a porcine model. *Thrombosis and Haemostasis*.

[76] Chandrasekar B, Nattel S, Tanguay JF (2001). Coronary artery endothelial protection after local delivery of 17*β*-estradiol during balloon angioplasty in a porcine model: a potential new pharmacologic approach to improve endothelial function. *Journal of the American College of Cardiology*.

[77] Chandrasekar B, Tanguay JF (2000). Local delivery of 17-beta-estradiol decreases neointimal hyperplasia after coronary angioplasty in a porcine model. *Journal of the American College of Cardiology*.

[78] Novensa L, Selent J, Pastor M, Sandberg K, Heras M, Dantas AP (2010). Equine estrogens impair nitric oxide production and endothelial nitric oxide synthase transcription in human endothelial cells compared with the natural 17*β*-estradiol. *Hypertension*.

[79] Sobrino A, Mata M, Laguna-Fernandez A (2009). Estradiol stimulates vasodilatory and metabolic pathways in cultured human endothelial cells. *PloS one*.

[80] Sobrino A, Oviedo PJ, Novella S (2010). Estradiol selectively stimulates endothelial prostacyclin production through estrogen receptor-*α*. *Journal of Molecular Endocrinology*.

[81] Golding EM, Kepler TE (2001). Role of estrogen in modulating EDHF-mediated dilations in the female rat middle cerebral artery. *American Journal of Physiology—Heart and Circulatory Physiology*.

[82] Novella S, Dantas AP, Segarra G (2010). Gathering of aging and estrogen withdrawal in vascular dysfunction of senescent accelerated mice. *Experimental Gerontology*.

[83] Davidge ST, Zhang Y (1998). Estrogen replacement suppresses a prostaglandin H synthase-dependent vasoconstrictor in rat mesenteric arteries. *Circulation Research*.

[84] David FL, Carvalho MH, Cobra AL (2001). Ovarian hormones modulate endothelin-1 vascular reactivity and mRNA expression in DOCA-salt hypertensive rats. *Hypertension*.

[85] Kip KE, Marroquin OC, Shaw LJ (2005). Global inflammation predicts cardiovascular risk in women: a report from the women’s ischemia syndrome evaluation (WISE) study. *American Heart Journal*.

[86] Sumino H, Ichikawa S, Ohyama Y (2005). Effect of transdermal hormone replacement therapy on the monocyte chemoattractant protein-1 concentrations and other vascular inflammatory markers and on endothelial function in postmenopausal women. *American Journal of Cardiology*.

[87] Friedrich EB, Clever YP, Wassmann S, Hess C, Nickenig G (2006). 17*β*-estradiol inhibits monocyte adhesion via down-regulation of Rac1 GTPase. *Journal of Molecular and Cellular Cardiology*.

[88] Störk S, Baumann K, von Schacky C, Angerer P (2002). The effect of 17*β*-estradiol on MCP-1 serum levels in postmenopausal women. *Cardiovascular Research*.

[89] Störk S, von Schacky C, Angerer P (2002). The effect of 17*β*-estradiol on endothelial and inflammatory markers in postmenopausal women: a randomized, controlled trial. *Atherosclerosis*.

[90] Arenas IA, Armstrong SJ, Xu Y, Davidge ST (2006). Tumor necrosis factor-*α* and vascular angiotensin II in estrogen-deficient rats. *Hypertension*.

[91] Faulds MH, Zhao C, Dahlman-Wright K, Gustafsson JA (2012). The diversity of sex steroid action: regulation of metabolism by estrogen signaling. *Journal of Endocrinology*.

[92] Louet JF, LeMay C, Mauvais-Jarvis F (2004). Antidiabetic actions of estrogen: insight from human and genetic mouse models. *Current Atherosclerosis Reports*.

[93] Bonds DE, Lasser N, Qi L (2006). The effect of conjugated equine oestrogen on diabetes incidence: the women’s health initiative randomised trial. *Diabetologia*.

[94] Margolis KL, Bonds DE, Rodabough RJ (2004). Effect of oestrogen plus progestin on the incidence of diabetes in postmenopausal women: results from the women’s health initiative hormone trial. *Diabetologia*.

[95] Rossouw JE, Anderson GL, Prentice RL (2002). Risks and benefits of estrogen plus progestin in healthy postmenopausal women: principal results from the women’s health initiative randomized controlled trial. *The Journal of the American Medical Association*.

[96] Furberg CD, Vittinghoff E, Davidson M (2002). Subgroup interactions in the heart and estrogen/progestin replacement study: lessons learned. *Circulation*.

[97] Manson JAE, Hsia J, Johnson KC (2003). Estrogen plus progestin and the risk of coronary heart disease. *The New England Journal of Medicine*.

[98] Stamataki KE, Spina J, Rangou DB, Chlouverakis CS, Piaditis GP (1996). Ovarian function in women with non-insulin dependent diabetes mellitus. *Clinical Endocrinology*.

[99] Ding EL, Song Y, Malik VS, Liu S (2006). Sex differences of endogenous sex hormones and risk of type 2 diabetes: a systematic review and meta-analysis. *The Journal of the American Medical Association*.

[100] Harman SM (2006). Estrogen replacement in menopausal women: recent and current prospective studies, the WHI and the KEEPS. *Gender Medicine*.

[101] Kim J, Kim JY, Song KS (2007). Epigenetic changes in estrogen receptor *β* gene in atherosclerotic cardiovascular tissues and in-vitro vascular senescence. *Biochimica et Biophysica Acta*.

[102] Novensa L, Novella S, Medina P, Segarra G, Castillo N (2011). Aging negatively affects estrogens-mediated effects on nitric oxide bioavailability by shifting ER*α*/ER*β* balance in female mice. *PLoS ONE*.

[103] Novella S, Heras M, Hermenegildo C, Dantas AP (2012). Effects of Estrogen on vascular inflammation: a matter of timing. *Arteriosclerosis, Thrombosis, and Vascular Biology*.

[104] Ortmann J, Veit M, Zingg S (2011). Estrogen receptor-*α* but not -*β* or GPER inhibits high glucose-induced human VSMC proliferation: potential role of ROS and ERK. *Journal of Clinical Endocrinology and Metabolism*.

[105] Cignarella A, Bolego C, Pelosi V (2009). Distinct roles of estrogen receptor-*α* and *β* in the modulation of vascular inducible nitric-oxide synthase in diabetes. *Journal of Pharmacology and Experimental Therapeutics*.

[106] Cignarella A, Minici C, Bolego C (2006). Potential pro-inflammatory action of resveratrol in vascular smooth muscle cells from normal and diabetic rats. *Nutrition, Metabolism and Cardiovascular Diseases*.

[107] Nilsson S, Gustafsson JA (2011). Estrogen receptors: therapies targeted to receptor subtypes. *Clinical Pharmacology and Therapeutics*.

[108] Barros RP, Gustafsson JA Estrogen receptors and the metabolic network. *Cell Metabolism*.

